# Incidental nodal irradiation in patients with esophageal cancer undergoing (chemo)radiation with 3D-CRT or VMAT

**DOI:** 10.1038/s41598-022-26641-w

**Published:** 2022-12-25

**Authors:** David Paul Peschel, Mathias Düsberg, Jan C. Peeken, Jan Christian Kaiser, Kai Joachim Borm, Katharina Sommer, Stephanie E. Combs, Stefan Münch

**Affiliations:** 1 Department of Radiation Oncology, Klinikum Rechts Der Isar, Technical University Munich (TUM), Ismaninger Str. 22, 81675 Munich, Germany; 2 Institute of Radiation Medicine (IRM), Helmholtz Zentrum München (HMGU), Ingolstädter Landstraße 1, 85764 Oberschleißheim, Germany; 3 German Cancer Consortium (DKTK), Partner Site Munich, Munich, Germany

**Keywords:** Oesophageal cancer, Radiotherapy

## Abstract

The extent of elective nodal irradiation (ENI) in patients undergoing definitive chemoradiotherapy (dCRT) for esophageal squamous cell carcinoma (ESCC) remains unclear. The aim of this dosimetric study was to evaluate the extent of incidental nodal irradiation using modern radiation techniques. A planning target volume (PTV) was generated for 30 patients with node-negative esophageal carcinoma (13 cervical/upper third, 7 middle third, 10 lower third/abdomen). Thereby, no elective nodal irradiation (ENI) was intended. Both three-dimensional conformal radiotherapy (3D-CRT) and volumetric-modulated arc therapy (VMAT) treatment plans (50 Gy in 25 fractions) were calculated for all patients. Fifteen nodal stations were contoured according to the definitions of the AJCC and investigated in regard to dosimetric parameters. Compared to 3D-CRT, VMAT was associated with lower dose distribution to the organs at risk (lower D_mean_, V20 and V30 for the lungs and lower D_mean_ and V30 for the heart). For both techniques, the median D_mean_ surpassed 40 Gy in 12 of 15 (80%) nodal stations. However, VMAT resulted in significantly lower D_means_ and equivalent uniform doses (EUD) compared to 3D-CRT for eight nodal stations (1L, 2L, 2R, 4L, 7, 8L, 10L, 15), while differences did not reach significance for seven nodal station (1R, 4R, 8U, 8M, 10R, 16). For dCRT of ESCC, the use of VMAT was associated with significantly lower median (incidental) doses to eight of 15 regional lymph node areas compared to 3D-CRT. However, given the small absolute differences, these differences probably do not impair (regional) tumor control rates.

## Introduction

Globally, esophageal cancer is the eighth most common cancer with over 600,000 newly diagnosed cases in 2020. Furthermore, with an estimated 544,000 deaths in 2020, esophageal cancer results in the sixth most common cancer related deaths worldwide^1^.

While patients with locally advanced esophageal squamous cell carcinoma (ESCC) are mostly treated with neoadjuvant chemoradiotherapy and surgery (nCRT + S), patients with locally advanced esophageal adenocarcinoma are usually treated with perioperative chemotherapy and surgery, or with nCRT + S, such as ESCC^2,3^. For both subtypes, this multidisciplinary approach leads to higher R0 resection rates and higher survival rates compared to surgery alone^4–7^. For patients with unresectable tumors, or patients who are unfit or unwilling to undergo surgery, definitive chemoradiotherapy is the treatment of choice.

Even though radiotherapy is commonly used in the treatment of ESCC, optimal treatment volumes are still heavily debated. One issue of this debate is whether elective nodal irradiation (ENI) can be safely omitted for ESCC patients undergoing dCRT. Two recent phase III trials used involved-field irradiation (IFI) for patients undergoing nCRT^4,8^. This means that in these studies the PTV was generated by adding specific longitudinal and radial safety margins around the gross tumor volume, but there was no specific inclusion of the lymphatic pathways. In contrast to that, ENI is still recommended for patients undergoing dCRT^2,9^.

Two recent meta-analyses found no significant differences between ENI and IFI in terms of local tumor control and overall survival (OS) in patients undergoing dCRT^10,11^. However, most patients were treated with three-dimensional conformal radiation therapy (3D-CRT). This is important to keep in mind, since 3CD-CRT has been the standard radiation technique for many years, but nowadays modern radiation techniques, such as intensity modulated radiation therapy (IMRT) and volumetric-modulated arc therapy (VMAT), are increasingly used in clinical routines. Compared to 3D-CRT, these techniques are characterized by a higher dose conformity and sharper dose gradients around the target volume^12–14^, which might affect the extent of incidental nodal irradiation.

Therefore, the purpose of this study is to compare the extent of incidental nodal irradiation between 3D-CRT and VMAT in patients with esophageal cancer undergoing IFI.

## Patients and methods

### Patients

Thirty patients who underwent chemoradiation for esophageal cancer in our department between 2011 and 2017 were retrospectively analyzed using medical records. All patients had pathologically confirmed ESCC or adenocarcinoma of the esophagogastric junction (AEG). Because we wanted to analyze incidental nodal irradiation, patients with macroscopic lymph node metastases that might lead to anatomic changes in the mediastinum were excluded from this study. For all patients, tumor location was classified according to the distance between the dental arch and the epicenter of the tumor. Clinical characteristics of all patients are summarized in Table 1.

**Table 1:** Patients’ baseline and tumor parameters.

**Age, (years)**	
Median (range)	70 (45–87)
Male sex, n (%)	20 (67)
**Clinical T-stage, n** (%)	
T2	4 (13)
T3	25 (83)
T4	1 (3)
**Tumor length, (cm)**	
Median (range)	5.9 (2.5–11.3)
**Tumor location, n (%)**	
Upper third/cervical	13 (43)
Middle third	7 (23)
Lower third/abdominal	10 (33)

### Target volume delineation

In all patients, treatment planning CT was done in supine position with a slice thickness of 3 mm using the Siemens Somatom Emotion 16 device (Siemens Healthineers, Erlangen, Germany).

Delineation of the planning target volume (PTV) was performed using the Varian Eclipse software (Medical Systems, Palo Alto, CA, USA). The primary tumor was delineated using all available information including CT, endoscopy, and positron-emission tomography CT (PET-CT), if available. Planning target volume (PTV) was defined by adding a proximal and distal margin of 4 cm along the esophagus, and an axial margin of 1.5 cm. In case of tumor extension into the stomach, a distal margin of 3 cm was alternatively used. In addition, radial overlap of the PTV with the heart and liver was limited to 1 cm.

### Treatment planning

For each patient, both a VMAT and a 3D-CRT treatment plan were calculated and optimized by an experienced medical physicist. For all patients the prescribed dose was 50 Gy in 25 fractions, normalized to the median PTV dose. To ensure a homogenous dose distribution within the PTV it was also aimed to cover the PTV by at least 95% of the prescribed dose, while not exceeding 107% of the prescribed dose. Planning goals regarding organs at risks were based on current NCCN guidelines^2^ and defined as summarized below.

Maximum spinal cord dose ≤ 45 Gy, mean whole lung dose < 15 Gy, V20 (whole lung) < 30%, V30 (whole lung) < 15%, mean heart dose < 20 Gy, V30 (heart) < 30%.

3D-CRT plans were designed using the Varian Eclipse Treatment Planning System (TPS) 15.6 (Varian Medical Systems, Palo Alto, CA, USA) for a linear accelerator (Trilogy, Varian medical System, Palo Alto, CA, USA). There were no compulsory restrictions regarding photon energy, the number of assigned fields, their arrangement and collimator angles, nor for the usage of wedge filters. VMAT plans were optimized by the Varian Photon Optimizer 15.1.51 (Varian Medical Systems, Palo Alto, CA, USA) using collimator angles between 5° and 15°. Treatment plans were calculated using the AAA 13.026 algorithm. All plans were reviewed by an experienced radiation oncologist regarding clinical feasibility.

### Treatment plans

**Figure 1:**
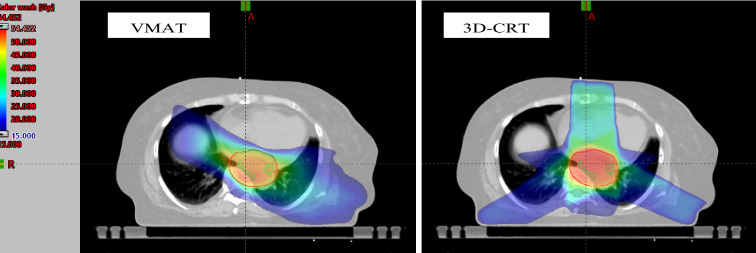
Dose distribution of the VMAT (left) and the 3D-CRT (right) plans of an exemplary patient in transversal view. Circled in red: PTV; circled in green: lower thoracic paraesophageal lymph nodes (8L).

3D-CRT plans were generated with a median of 5 (range 4–8) coplanar beams, using 6 or 15 megaelectron volt (MV). Most of the monitor units (MUs) were delivered by left-posterior fields around 220°, right-posterior fields around 130° and an anterior field of 0° gantry rotation. For all VMAT plans, two complete arcs were used to deliver the planned dose, operating with photon energies of 6 MV or 15 MV. Regarding photon energies, 6-MV photons were used for 4 treatment plans (3D-CTRT) and 9 treatment plans (VMAT), while 15-MV photons were used for 19 treatment plans (3D-CRT) and 21 treatment plans (VMAT), respectively. Both 6-MV photons and 15-MV photons were used for 7 3D-CRT treatment plans. Thereby, the same photon energy was used for 3D-CRT and VMAT plans in 21 patients (70%). Figure 1 displays the dose distribution of 3D-CRT and VMAT plans using an exemplary patient.

### Nodal stations

For all patients, the following 15 regional nodal station were delineated by the same person according to the 8th edition of AJCC Cancer staging manual^15^:

1L: left lower cervical paratracheal nodes, 1R: right lower cervical paratracheal nodes, 2L: left upper paratracheal nodes, 2R: right upper paratracheal nodes, 4L: left lower paratracheal nodes, 4R: right lower paratracheal nodes, 7: subcarinal nodes, 10L: left hilar nodes, 10R: right hilar nodes 8U: upper thoracic paraesophageal lymph nodes 8M: middle thoracic paraesophageal lymph nodes 8L: lower thoracic paraesophageal lymph nodes 15: diaphragmatic nodes 16: paracardial nodes, 17: left gastric nodes.

Because (incidental) dose distribution to the nodal regions is strongly affected by the localization and lengths of the primary tumor and the corresponding PTV, nodal stations cranial or caudal of the PTV were excluded from the analysis in each patient. In addition, the proportions of all remaining lymph node stations, that were located cranial or caudal of the PTV were also excluded from the analysis. For each nodal region, the mean dose (D_mean_) and the percentage of volume receiving more than 20 Gy (V20), 30 Gy (V30) and 40 Gy (V40) were computed. Additionally, the equivalent uniform dose (EUD) was calculated for each region. EUD is a quantity to compare inhomogeneous dose distributions by converting them into a homogeneous dose distribution, which would cause the same biological effect as the actual inhomogeneous dose distribution^16^.

The following formula was used to calculate the EUD:

$$\mathrm{EUD}={\left(\frac{1}{N} \sum_{i=1}^{N}{\mathrm{D}}_{i}^{a}\right)}^\frac{1}{a}$$

(*N*—number of voxels in the examined anatomic structure; *D*—absorbed doses in the voxel “*i*”; *a*—tissue specific parameter that influences how minimal or maximal doses are adjusted to the EUD). Thereby, a = − 5 was used as described by Ji et al.^17^.

### Statistics

The statistical analysis was performed using SPSS 27.0.1.0 software (SPSS Inc, Chicago, IL, USA). Since the observed data did not have a gaussian distribution, the Wilcoxon signed-rank test for paired samples was used. The statistical significance was considered at a p value of < 0.05.

### Ethics approval and consent to participate

The ethical committee of the Technical University of Munich has approved the retrospective study protocol (ethical vote N° 134/21 S-EB). All patients gave their written informed consent for radiotherapy. All methods were performed in accordance with the relevant guidelines and regulations.

## Results

### Patients

The median age of the 30 patients of our study was 70 years and 67% of them were male. 87% of the patients had locally advanced tumors (T3/4). The median length of the tumors was 5.9 cm. In 43% of all cases, the tumor was located in the cervical esophagus or upper third of the thoracic esophagus, while it was found in the middle third in 23% of patients. The tumor of the remaining 10 patients (33%) was found in the lower third of the thoracic esophagus or the abdomen.

### Organs at risk

Table 2 shows the dose distribution to the organs at risk for 3D-CRT and VMAT. While no significant difference between VMAT and 3D-CRT was seen for spinal cord maximum dose, VMAT was associated with a lower dose distribution to the lungs regarding mean dose (9.6 Gy ± 4.3 Gy (3D-CRT) vs. 8.7 Gy ± 3.9 Gy (VMAT), p < 0.001), V20 (15.4% ± 8.9% (3D-CRT) vs. 8.9% ± 7.5% (VMAT), p < 0.001) and V30 (4% ± 3.5% (3D-CRT) vs. 2.6% ± 2.9% (VMAT), p < 0.001). Also, VMAT was associated with a lower dose distribution to the heart regarding mean dose (11.7 Gy ± 10.1 Gy (3D-CRT) vs. 9.7 Gy ± 8.2 Gy (VMAT), p < 0.001) and V30 (9.5% ± 11.4% (3D-CRT) vs. 5.7% ± 9.4% (VMAT), p < 0.001). In contrast no significant differences were seen regarding V5 for the lungs.

**Table 2:** Dose distribution to the organs at risk.

	3D-CRT Median ± SD	VMAT Median ± SD	p value
Spinal cord D_max_ (Gy)	35.5 ± 11.9	34 ± 9.6	0.516
Lung D_mean_ (Gy)	9.6 ± 4.3	8.7 ± 3.9	** < 0.001**
Lung V5 (%)	51.3 ± 23.7	50.8 ± 24.8	0.761
Lung V20 (%)	15.4 ± 8.9	8.9 ± 7.5	** < 0.001**
Lung V30 (%)	4 ± 3.5	2.6 ± 2.9	** < 0.001**
Heart D_mean_ (Gy)	11.7 ± 10.1	9.7 ± 8.2	** < 0.001**
Heart V30 (%)	9.5 ± 11.4	5.7 ± 9.4	** < 0.001**

*SD* standard deviation, *3D-CRT* three-dimensional conformal radiotherapy, *D*_*max*_ maximum dose, *D*_*mean*_ mean dose, *V05–V30* volume receiving more than 5–30 Gy, *VMAT* volumetric-modulated arc therapy. Significant values are in bold.

### (Incidental) nodal dose

Tables 3, 4, and 5 display computed mean doses, EUD-, V20-, V30- and V40-values for all examined lymph node stations for both 3D–CRT and VMAT plans. In summary, VMAT resulted in significantly lower D_mean_ and EUD compared to 3D-CRT in eight (of 15) investigated lymph node stations (1L, 2L, 2R, 4L, 7, 8L, 10L and 15).

**Table 3:** Dose parameters for cervical/upper thoracic nodal stations.

	3D-CRT Median ± SD	VMAT Median ± SD	p value	3D-CRT Median ± SD	VMAT Median ± SD	p value
	**1L (n = 14)**			**1R (n = 14)**		
D_mean_ (Gy)	48.8 ± 6.5	47.2 ± 4.9	**0.025**	48.7 ± 5.8	48.8 ± 6.4	0.358
EUD (Gy)	48.4 ± 7.7	45.3 ± 6.4	**0.017**	48.4 ± 6.7	47.7 ± 7.4	0.326
V20 (%)	100 ± 0	100 ± 0	1.000	100 ± 0	100 ± 0.1	1.000
V30 (%)	100 ± 15.3	100 ± 7	0.438	100 ± 3.8	100 ± 12.1	0.250
V40 (%)	98.7 ± 35.6	86 ± 31.7	0.105	99.9 ± 33.4	95.5 ± 31.9	0.297
	**2L (n = 15)**			**2R (n = 16)**		
D_mean_ (Gy)	51 ± 1.1	50.1 ± 0.9	** < 0.001**	48.1 ± 5.1	46.7 ± 5.4	**0.039**
EUD (Gy)	51 ± 1.2	50 ± 1.3	** < 0.001**	46.5 ± 7	43.2 ± 6.8	**0.043**
V20 (%)	100 ± 0	100 ± 0	1.000	100 ± 3.2	100 ± 0	1.000
V30 (%)	100 ± 0	100 ± 0.1	1.000	100 ± 13.2	100 ± 14.5	0.109
V40 (%)	100 ± 0.5	100 ± 2.7	**0.031**	97.2 ± 21	85.2 ± 25.2	**0.008**
	**4L (n = 20)**			**4R (n = 21)**		
D_mean_ (Gy)	49.1 ± 6.5	48.7 ± 7.2	**0.006**	39.3 ± 11.1	36 ± 8.8	0.094
EUD (Gy)	48.9 ± 9	48.3 ± 8.9	**0.021**	33.6 ± 11.6	30 ± 9.2	0.320
V20 (%)	100 ± 8	100 ± 4.3	0.625	99.5 ± 31.1	99.4 ± 17.6	0.173
V30 (%)	100 ± 13.8	100 ± 25.6	**0.016**	84.7 ± 37	80.7 ± 33.5	0.051
V40 (%)	99.9 ± 29.9	99.5 ± 30.9	** < 0.001**	57.4 ± 36.5	33.8 ± 34.8	** < 0.001**
	**8U (n = 21)**					
D_mean_ (Gy)	49.7 ± 1.7	49.9 ± 1.9	0.257			
EUD (Gy)	49.5 ± 2.2	49.9 ± 4.1	0.615			
V20 (%)	100 ± 0	100 ± 0.3	1.000			
V30 (%)	100 ± 0.1	100 ± 4.1	0.148			
V40 (%)	100 ± 5.8	99.7 ± 7.7	** < 0.001**			

*SD* standard deviation, *3D-CRT* three-dimensional conformal radiotherapy, *D*_*mean*_ mean dose, *EUD* equivalent uniform dose, *n* number of included patients, *V20–V40* volume receiving more than 20–40 Gy, *VMAT* volumetric-modulated arc therapy. Significant values are in bold.

**Table 4:** Dose parameters for middle thoracic nodal stations.

	3D-CRT Median ± SD	VMAT Median ± SD	p value	3D-CRT Median ± SD	VMAT Median ± SD	p value
	**10L (n = 16)**			**10R (n = 14)**		
D_mean_ (Gy)	26.4 ± 7.5	22.5 ± 5.9	**0.011**	24.4 ± 11.4	25 ± 12.2	0.058
EUD (Gy)	24 ± 7.7	21.5 ± 5.7	**0.029**	23.3 ± 11.5	24.4 ± 12.4	0.091
V20 (%)	91.3 ± 34.9	78.4 ± 38.3	0.176	98.4 ± 28.3	97.7 ± 42.7	0.074
V30 (%)	26.6 ± 27.2	2.4 ± 16.8	**0.002**	10.2 ± 41.4	2.2 ± 41	0.438
V40 (%)	0.4 ± 19.5	0 ± 4.6	**0.019**	0 ± 42.4	0 ± 42.1	0.375
	**7 (n = 22)**			**8 M (n = 22)**		
D_mean_ (Gy)	46.1 ± 6.1	44.4 ± 7.5	**0.009**	49.2 ± 1.5	49.1 ± 1.5	0.799
EUD (Gy)	44.2 ± 7.2	38 ± 10.1	** < 0.001**	48.5 ± 4.2	47.8 ± 3.6	0.633
V20 (%)	100 ± 0	100 ± 7.1	**0.016**	100 ± 0.3	100 ± 0.1	1.000
V30 (%)	99.9 ± 15.7	96.4 ± 24.6	**0.002**	100 ± 3.1	99.9 ± 2	0.217
V40 (%)	90.6 ± 33.4	75.1 ± 34.7	**0.003**	98.6 ± 5.5	97.4 ± 6.6	**0.003**

*SD* standard deviation, *3D-CRT* three-dimensional conformal radiotherapy, *D*_*mean*_ mean dose, *EUD* equivalent uniform dose, *n* number of included patients, *V20 – V40* volume receiving more than 20 – 40 Gy, *VMAT* volumetric-modulated arc therapy. Significant values are in bold.

**Table 5:** Dose parameters for lower thoracic/abdominal nodal stations.

	3D-CRT Median ± SD	VMAT Median ± SD	p value	3D-CRT Median ± SD	VMAT Median ± SD	p value
	**15 (n = 14)**			**16 (n = 13)**		
D_mean_ (Gy)	48.4 ± 3.2	46.5 ± 4	**0.020**	49.3 ± 5.7	49.9 ± 6.1	0.376
EUD (Gy)	46 ± 7.2	40.1 ± 10	**0.049**	48.5 ± 11.6	49.6 ± 10.9	0.893
V20 (%)	100 ± 1.8	100 ± 4.5	0.469	100 ± 5	100 ± 5.3	0.813
V30 (%)	99.5 ± 3.7	95.8 ± 9.7	**0.042**	100 ± 12.8	100 ± 16.8	0.063
V40 (%)	92.3 ± 10.7	85.9 ± 16.4	**0.001**	100 ± 21.4	99.7 ± 23.7	0.469
	**17 (n = 10)**			**8L (n = 17)**		
D_mean_ (Gy)	47.3 ± 10.4	48.6 ± 11.3	0.084	50.6 ± 1.9	49.5 ± 2.2	**0.001**
EUD (Gy)	46.2 ± 11.7	47 ± 12	0.084	50.1 ± 2.7	49.2 ± 3.9	** < 0.001**
V20 (%)	100 ± 22.4	100 ± 24.2	0.500	100 ± 0	100 ± 0.1	0.500
V30 (%)	99.8 ± 31.9	100 ± 35.6	0.625	100 ± 0.5	100 ± 1.6	**0.016**
V40 (%)	95.5 ± 39.4	93 ± 40.1	0.313	99.5 ± 2.9	98.9 ± 9.6	**0.042**

± standard deviation, *3D-CRT* three-dimensional conformal radiotherapy, *D*_*mean*_ mean dose, *EUD* equivalent uniform dose, *n* number of included patients, *V20–V40* volume receiving more than 20–40 Gy, *VMAT* volumetric-modulated arc therapy. Significant values are in bold.

In contrast, no significant difference regarding D_mean_ and EUD between 3D-CRT and VMAT was seen for all other lymph node stations (1R, 4R, 8U, 8M, 10R, 16 and 17).

Regarding the dose parameters V20, V30, and V40 in five nodal stations (2L, 2R, 4R, 8U and 8M) VMAT was associated with significantly lower V40. In four more nodal stations (4L, 8L, 10L and 15) VMAT was linked to significantly lower V30 and V40. For subcarinal nodes (7) V20, V30 and V40 was significantly reduced by VMAT. However, for five of the investigated lymph node stations (1L, 1R, 10R, 16 and 17) no significant differences for V20, V30 or V40 were seen between 3D-CRT and VMAT.

**Figure 2:**
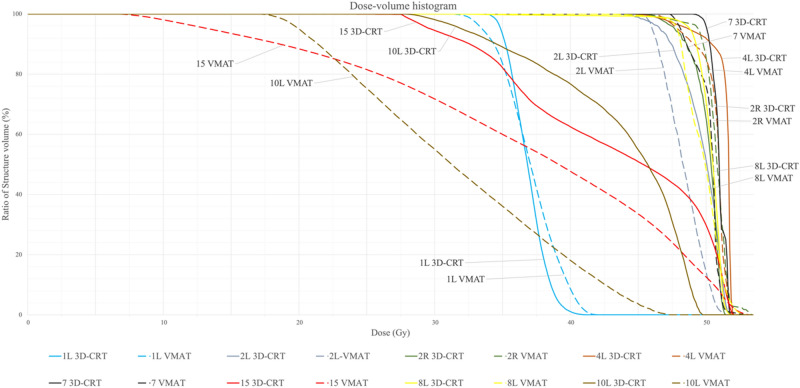
Comparison of DVHs of a representative case: for reasons of clarity, only nodal stations with significant differences between 3D-CRT and VMAT regarding D_mean_ and EUD are displayed. VMAT (dashed line); 3D-CRT (solid line).

A representative DVH for VMAT and 3D-CRT plans is shown in Fig. 2

## Discussion

The aim of this study was to compare the extent of incidental nodal irradiation between 3D-CRT and VMAT for patients with esophageal cancer undergoing IFI. Our study demonstrated that a considerable amount of incidental dose is delivered to the regional lymph node stations for both 3D-CRT and VMAT. However, VMAT was associated with significant lower mean doses and EUDs in 8 out of 15 investigated lymph node stations compared to 3D-CRT (1L, 2L, 2R, 4L, 7, 10L, 8L, 15).

For both investigated radiation techniques, incidental dose distribution to nodal stations was strongly affected by their radial distance to the PTV. Thereby, incidental nodal irradiation declined with increasing distance to the PTV. Nodal stations immediately adjacent to the esophagus (8U, 8M, 8L, 2L) were exposed to irradiation close to the prescribed dose, whereas the hilar nodes, located the furthest away from the esophagus, were irradiated the least. Spatial distance heavily effecting nodal irradiation was previously described by Ji et al.^17^ in a study that investigated incidental nodal irradiation using 3D-CRT with a prescribed dose of 60 Gy. For upper thoracic ESCC treated with IFI, the median EUD to the cervical paraesophageal nodes was 61.1 Gy (range 2.2–65.5 Gy), while the lowest median EUDs were seen for the supraclavicular nodes (median EUD: 15.2 Gy, range 2.1–66.2 Gy), having the greatest radial distance to the esophagus. Considering that there are small differences in defining the different lymph node regions between the Japan esophageal society^18^ (used by Ji et al.) and the AJCC, and that not only parts of lymph node stations on the same height as the PTV were investigated, which might explain the wider range of measured EUDs; our study was able to confirm the findings of Ji et al. for both 3D-CRT and VMAT.

For bilateral nodal stations, the left-side tended to be exposed to a greater incidental dose than the right counter sides, regardless of the applied technique. This was seen for the upper paratracheal nodes (2L and 2R) and the lower paratracheal nodes, while similar dose distributions were seen for the left and right cervical paratracheal nodes (1L and 1R) as well as the hilar nodes (10L and 10R). These results were in line with the results of the previously mentioned study by Ji et al.^17^. In their study, incidental dose (EUD) to the left tracheobronchial nodes was higher than to the right tracheobronchial nodes. It should be mentioned that the anatomic definition of the tracheobronchial nodes in their study is similar to the anatomic definition of the lower paratracheal nodes (4L and 4R) in our study. Different observations were published in a study by Zhang et al.^19^ investigating incidental nodal irradiation in patients with esophageal cancer using different radiation techniques. In their study, incidental doses (EUD and V40) to the right tracheobronchial nodes were higher than incidental doses to the left tracheobronchial nodes for 3D-CRT and VMAT. It should be noted that some methodological weaknesses can be found in this study. There is a lack of information on normal tissue constraints, the delineation of the PTVs, and the applied parameters calculating the EUDs. A possible explanation for left nodal stations being exposed to a higher incidental dose than their right counter side is the course of the esophagus being placed centrally in the neck moving further to the left-hand side in the chest. Since Hilar nodes are placed cranial to the heart and surrounded by lung tissue, given constraints and VMATs superiority in fulfilling these constraints may have had an additional influence on dose distribution to these stations.

In 8 out of 15 investigated nodal stations (1L, 2L, 2R, 4L, 7, 10L, 8L, and 15), VMAT was associated with significantly lower mean doses and EUDs compared to 3D-CRT. In addition, V40 was significantly reduced by VMAT in 10 out of 15 nodal stations (2L, 2R, 4L, 4R, 7, 8U, 8M, 8L, 10L and 15), while VMAT was not associated with a significant higher dose distribution compared to 3D-CRT for any of the analyzed lymph node stations, nor any of the analyzed dose parameters (see Tables 3, 4, 5). Our findings differ at least partly from the previously mentioned work by Zhang et al. In patients with upper-thoracic tumor localization, VMAT was also associated with a significantly lower incidental dose (EUD) to the pretracheal lymph nodes (106pre) and the left tracheobronchial nodes (106tb-L) compared to 3D-CRT, while VMAT was associated with a significantly higher incidental dose to the left tracheobronchial nodes (106tb-L), the bilateral recurrent nerve lymph nodes (106recR and 106recL), the subcarinal lymph nodes (107), the bilateral pulmonary ligament nodes (112pul-R and 112pul-L), and the thoracic paraaortic nodes (112ao) in patients with middle and lower thoracic tumor localization^19^. Zhang et al. explicated the inconsistency of their results by the applied beam arrangement for the 3D-CRT plans. They state that for patients with middle or lower thoracic tumor localization the majority of lymph nodes were out of the irradiation fields. Since the same beam arrangement was used for upper- and middle-thoracic tumor localization (two parallel-opposed oblique fields and anteroposterior–posterioranterior fields) in their study, we were not able to comprehend nor reproduce these findings.

In general, compared to 3D-CRT, the usage of VMAT results in an improvement of target coverage and dose conformity, leading to a better sparing of normal tissue, which is shown by various studies^12–14^, including the previously mentioned study by Zhang et al.^19^. Our results confirm the superiority of VMAT regarding the sparing of OARs (see Fig. 1, see Table 2).

Considering this, it is plausible that VMAT was not associated with a higher dose distribution compared to 3D-CRT for any of the analyzed lymph node station nor any of the analyzed dose parameters.

Nevertheless, differences between the two radiation techniques regarding D_mean_ and EUD did not reach significance in all of the 15 investigated nodal stations. Moreover, it should be noted that the absolute differences between the techniques were relatively small, only surpassing four Gray for the lymph nodes stations 7 and 15. This could at least partly be explained by the radial expansion from GTV to PTV, which leads to portions of the nodal stations being covered by the PTV. Therefore, the impact of the applied radiation technique on incidental nodal irradiation decreases for these stations (see Fig. 1), since the applied radiation technique only becomes relevant for portions of nodal stations outside of the PTV.

For ESCC patients treated with dCRT, a relevant pattern of failure is locoregional recurrences^20–22^. With advances in imaging and radiation techniques, treatment volumes in dCRT have decreased over time, but the necessity of ENI is still heavily debated. Regarding elective-nodal irradiation compared to involved-field irradiation, evidence-based medicine relies on mostly retrospective and monocentric trials applying mainly 3D-CRT^23–26^. Meta-analysis pooling of these trials concluded no improvement of OS nor regional tumor control for ENI compared to IFI, while associated to higher rates of esophageal and lung toxicity^10,11^.

Ji et al. investigated incidental nodal irradiation using 3D-CRT and concluded that D_means_ and EUDs for most high-risk nodal regions were greater than 40 Gy for a prescribed dose of 60 Gy^16^. In a study by Onozawa et al., including 102 patients with ESCC, a dosage of 40 Gy was delivered to the entire lymphatic drainage of the esophagus, resulting in only one case of elective nodal failure^27^. Hence, Ji et al. suggested similar tumor control and survival rates between ENI and IFI as a possible result of incidental nodal irradiation. In our study median D_means_ and EUDs were greater than 40 Gy for most of the investigated lymph node stations, even for a prescribed dose of 50 Gy. Using 3D-CRT only for the hilar nodes (10L and 10R) and for the right lower paratracheal nodes (4R), median D_mean_ and EUD were smaller than 40 Gy. In addition to this, using VMAT, the median EUD at the subcarinal nodes (EUD: 38 Gy ± 10.1 Gy) was also smaller than 40 Gy.

To the best of our knowledge, there is no study directly comparing ENI and IFI in dCRT for patients exclusively treated with VMAT or IMRT. If incidental nodal irradiation is indeed the likely reason for there being no significant difference observed between ENI and IFI for 3D-CRT, results from this study serve as a potential precursor for application of more advanced technologies such as IMRT or VMAT and as to whether or not they should be applied in the dCRT setting.

Differences in incidental nodal irradiation between 3D-CRT and VMAT were detected in this study. In the investigated stations, these detected differences in irradiation varied in their degree due to beam arrangement and anatomical location. However, firstly for both radiation techniques, most of the nodal regions are exposed to relevant doses of irradiation, presumably since most nodal region are placed inside of or close to the PTV. Secondly, compared to the absolute amount of nodal irradiation for IFI, the differences caused by the selected irradiation technique are relatively low. Given these small absolute differences, results of the mentioned meta-analysis comparing IFI and ENI using 3D-CRT should probably be transferable to the usage of VMAT.

We are fully aware of the limitations of transferring dosimetric results to practice. Clearly, retrospective or better prospective studies are necessary to compare ENI with IFI for dCRT using IMRT or VMAT. Due to its dosimetric nature, this study has some limitations. Although the defined nodal regions are based on metastatic distribution patterns for esophageal cancer, their meaning for the individual patient can always be questioned. Since differences between 3D-CRT and VMAT regarding the measured parameters were relatively small, greater sample sizes may presumably have been able to detect statistically significant differences between the investigated radiation techniques at all nodal stations. Furthermore, since only nodal stations that were placed on the same height as the PTV were investigated, our statements about occurring nodal irradiation at regions that are cranial or caudal to the PTV are limited. However, as no nodal GTV was applied in this study, our results are calculated rather conservatively. For nodal positive patients with an additional nodal GTV resulting in a larger planning volume, the occurring irradiation to uninvolved nodal regions is presumably higher. Another limitation affects the use of different and especially high-energy photons, because of the risk of inaccuracies in the calculated dose distribution in areas with large density variations like the lungs. However, for recent AAA algorithms these inaccuracies have been reduced compared to earlier versions and should not affect clinical results or conclusion of this study^28–30^.

## Conclusion

For dCRT of ESCC, the use of VMAT was associated with significantly lower median (incidental) doses to eight of 15 regional lymph node areas compared to 3D-CRT. However, given the small absolute differences, these differences probably do not impair (regional) tumor control rates.

## Data Availability

The datasets used and/or analysed during the current study available from the corresponding author on reasonable request.
